# Potential Triggers for Thrombocytopenia and/or Hemorrhage by the BNT162b2 Vaccine, Pfizer-BioNTech

**DOI:** 10.3389/fmed.2021.751598

**Published:** 2021-09-30

**Authors:** Yusuke Okada, Ryota Sakai, Marie Sato-Fitoussi, Marika Nodera, Shoichi Yoshinaga, Akiko Shibata, Takahiko Kurasawa, Tsuneo Kondo, Koichi Amano

**Affiliations:** Department of Rheumatology and Clinical Immunology, Saitama Medical Center, Saitama Medical University, Kawagoe, Japan

**Keywords:** BNT162b2, COVID-19, hemorrhage, immune thrombocytopenia, rheumatoid arthritis

## Abstract

Immune thrombocytopenia is an autoimmune disease that can cause bleeding in severe cases. Although available published data do not associate the BNT162b2 vaccine (Pfizer-BioNTech) with the risk of developing thrombocytopenia, the ChAdOx1 nCov-19 vaccine has raised concerns about its potential link with thrombosis and thrombocytopenia. We would like to clarify whether the BNT162b2 vaccine administration may interfere with pre-existing conditions and whether it may cause a risk of thrombocytopenia. Herein, we report three cases of post-vaccine thrombocytopenia among patients with rheumatoid arthritis (RA); one case in which a causal relationship cannot be ruled out with the BNT162b2 vaccine was officially announced. Furthermore, we reviewed reports of adverse events and death cases with a focus on thrombocytopenia and hemorrhages, following vaccination with BNT162b2 in Japan between February 17, 2021 and July 16, 2021, as reported by the Ministry of Health, Labour, and Welfare within the general population. The three cases in this report share the common features of old age, RA, chronic renal failure or hypertension, and pre-existing mild thrombocytopenia at baseline. A total of 746 death cases were reported during this time period, with death by bleeding accounting for 8.8% of the total deaths, of which 84.8% were cranial and statistically higher in young women than among elderly women. The risk-benefit ratio of the vaccine needs to be reconsidered based on high- and low-risk population types and ethnicity. To do so, the expansion of the pharmacovigilance system for BNT162b2 vaccination is urgently required worldwide.

## Introduction

The coronavirus disease 2019 (COVID-19) vaccine has been launched worldwide as an effective and efficient public health measure to control the pandemic. However, safety for vaccines must be carefully considered since adverse reactions after vaccine administration may vary according to race, ethnicity, and pre-existing conditions. Thrombosis and thrombocytopenia syndromes after ChAdOx1 nCov-19 (AstraZeneca) vaccination have rarely been reported but have strongly impacted the vaccination policy worldwide (1–4). On the contrary, the number of thrombocytopenia, including immune thrombocytopenia (ITP) cases after BNT162b2 (Pfizer-BioNTech) and mRNA-1273 (Moderna) vaccination is not greater than the number of ITP cases expected on an annual incidence rate according to the Vaccine Adverse Event Reporting System, as reported by Food and Drug Administration (5). Moreover, a Scotland study showed that no associations were found between thrombocytopenia, thrombosis, hemorrhage and the BNT162b2 vaccine (6). However, there are several reports of vaccine-induced thrombocytopenia (7–10). Frequency, risk, and underlying mechanisms of vaccine-associated thrombosis or thrombocytopenia in Japan are still unclear, with the major caveat that the reported adverse events are considered only temporally related to vaccine administration. In mid-February 2021, BNT162b2 vaccination for COVID-19 had begun for health care professionals in Japan, and on June 1st, vaccination opened for 12-year-old and older individuals. On July 7th, the Japanese Ministry of Health, Labour, and Welfare (MHLW) stated that causality is undeniable for a first case in which the BNT162b2 vaccination was a trigger for thrombocytopenia in a rheumatoid arthritis (RA) patient but did not mention vaccination as the determining factor for her later death. In this report, we present the details of this first case and two additional cases of elderly RA patients who displayed thrombocytopenia following BNT162b2 vaccination at our institute. In addition, we summarize the cases of post-vaccine thrombocytopenia or hemorrhage among the general population in Japan recorded by the MHLW and tentatively clarify the incidence, risk of developing these symptoms, and mechanisms underlying the emergence of vaccine-associated thrombocytopenia in the Japanese population.

## Methods

### MHLW Reports Analysis

We summarized the cases of thrombocytopenia, hemorrhage, and death after COVID-19 vaccination reported on the MHLW website. The MHLW detected a total of 746 deaths (male 379, female 364, unknown 3; <65 years 43, ≥65 years 698, unknown 5) including those whose causality with the vaccine administration remains to be elucidated, in a total of 57,401,937 doses (health care provider: 11,713,945 doses; others: 45,687,992 doses, including 48,294,827 doses for the elderly population, that is, <65 years 9,107,110 doses). At least one dose was administered to 35,650,101 persons by July 16th, which equals a prevalence of 12 deaths per million doses (11, 12). Sex and age-unspecified individuals, as well as unclassifiable individuals (for example, the individual is in his/her 60 s) were excluded from the analysis. The MHLW accepts reports from physicians, nurses, pharmacists, and manufacturers and distributors of cases as adverse event reports under the Pharmaceutical Affairs Law in Japan. The census from the earliest reports until the most recent report as of the last access date (July 27, 2021) were utilized in the analysis of cases presenting with thrombocytopenia. Document No.1-3-1 is a detailed report of cases of death from the 17th of February to the 16th of July. Documents No. 1-1-2-1, No. 1-2-2-1, and No. 1-2-3-1 are summaries and detailed reports of adverse events and deaths in cases following the administration of COVID-19 vaccines mapped by the MHLW from the 17th of February to the 11th of July. No. 1-2-3-1 included six time periods, from the 17th of February to the 2nd of May (file 1), the 3rd to the 16th of May (file 2), the 17th to the 30th of May (file 3), the 1st to the 13th of June (file 4), the 14th to the 27th of June (file 5), and the 27th to the 11th of July (file 6) (13). Search terms relating to “thrombocytopenia,” “immune thrombocytopenia,” “platelet count decreased,” and “hemorrhage” or “bleeding” in Japanese, as well as “platelets” and “PLT” in English were used to identify cases of interest. The noun “platelet” is composed of three Japanese characters “血” (blood) + “小” (small) + “板” (plate) = 血小板 (platelet). In order to avoid any overlooking due to eventual indention between the characters of a given word, each individual characters were searched in addition to the whole word (for example, “blood,” “small,” and “plate” were searched in addition to “platelet”). The same method was applied to all the other keywords. All cases were reported as lot, day of vaccination, age of the individuals, and description of events coincided were deemed to be reported in double and were thus excluded from the analysis. The diagnosis of thrombocytopenia was deemed by physicians, nurses, pharmacists, or field-related workers according to the Pharmaceutical Affairs Law. Thus, cases of thrombocytopenia reported without the mention of platelet counts were still inspected.

### Statistical Analysis

Statistical significance was determined by Pearson's chi squared test for independent samples in case of a 2 × 2 table. Differences were considered significant at *P* < 0.05, and all analyses were performed using JMP 15 (SAS Institute Inc., Cary, NC, USA).

## Results

### Case 1

An 80-year-old woman was diagnosed with RA in 2010 based on polyarthritis, high serum C-reactive protein levels, positive for anti-cyclic citrullinated peptide (CCP) antibody, and rheumatoid factor (RF). She was first treated with methotrexate (MTX), followed by a switch to golimumab (GLM) monotherapy due to chronic kidney disease (CKD). She was also taking epoetin beta pegol (the last dose of both was 12 days before vaccine administration) for renal anemia due to CKD stage G5A2 and daily levothyroxine sodium hydrate for Hashimoto's disease. Four days after the second dose of the BNT162b2 vaccine, headache, nausea, loss of consciousness, and purpura appeared, for which she was admitted to our emergency department. Blood tests revealed marked thrombocytopenia (0.8 × 10^4^/μL) despite a last count of 11.2 × 10^4^/μL, and brain computed tomography (CT) revealed subarachnoid hemorrhage. No cerebral aneurysm or apparent thrombosis was observed. Platelet counts were 0.3 × 10^4^/μL despite frequent platelet transfusion, and the patient died from intracranial re-bleeding 3 days after admission. There were no autoantibodies, including anti-nuclear antibody or anti-dsDNA antibody, but only platelet-associated immunoglobulin G (PA-IgG) was as high as 206 ng/10^7^ cells (standard value <46 ng/10^7^ cells).

### Case 2

A 77-year-old man was diagnosed with RA in 2006 with positive anti-CCP antibodies and RF; MTX was used but was changed to GLM in 2016 (the recent dose interval was 6–7 weeks; the last dose was 5 weeks before vaccine administration) and maintained in remission. The patient was receiving epoetin beta pegol and zinc acetate hydrate for hypertension and chronic renal failure. Four days after the first dose of the BNT162b2 vaccine, the patient developed left upper and lower limb paralysis and was rushed to the emergency room. The blood test revealed a platelet count of 5.6 × 10^4^/μL despite the last count of 11.1 × 10^4^/μL, and the brain CT showed right frontal lobe subcortical hemorrhage and subarachnoid hemorrhage in the central sulcus. There were no significant thrombotic findings. Transient thrombocytopenia recovered spontaneously after a few days, and the patient was transferred to the hospital for rehabilitation. The results of the PA-IgG test were negative.

### Case 3

A 79-year-old man diagnosed with RA in 2019 with positive anti-CCP antibodies and RF had a long history of hypertension. He developed primary ITP (PA-IgG negative) at the time of sarilumab induction and the latter was therefore substituted with prednisolone (PSL) 20 mg/day and intravenous immunoglobulin. Five days following the first dose of the BNT162b2 vaccine, the patient visited the outpatient clinic with no signs of bleeding but with a low platelet count of 4.7 × 10^4^/μL despite a last count of 15.2 × 10^4^/μL. The patient was deemed to have a flare of ITP, and PSL was increased from 9 to 15 mg/day, and tacrolimus 2 mg/day was added. At his firm request, a second vaccination was administered, after which the platelet count increased to 16.0 × 10^4^/μL. The PA-IgG level following the second vaccination was 75 ng/10^7^ cells.

**Figure 1:**
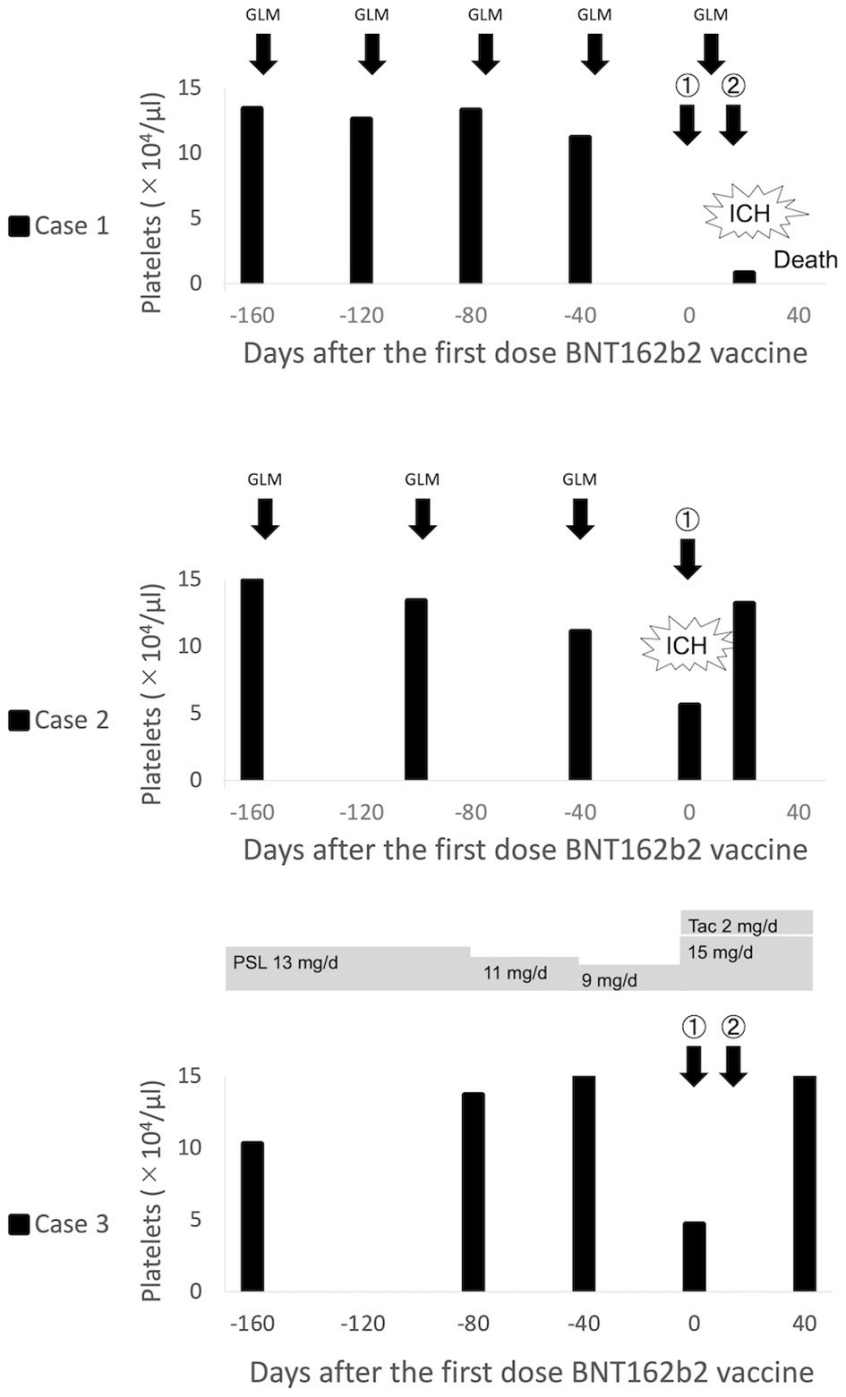
Clinical course of thrombocytopenia in patients with rheumatoid arthritis after BNT162b2 vaccination. ICH, Intracranial hemorrhage; GLM, Golimumab; PSL, Prednisolone; Tac, Tacrolimus.

The clinical course of all cases is presented in Figure 1.

### Review of Case Presentations in the MHLW Reports

**Figure 2:**
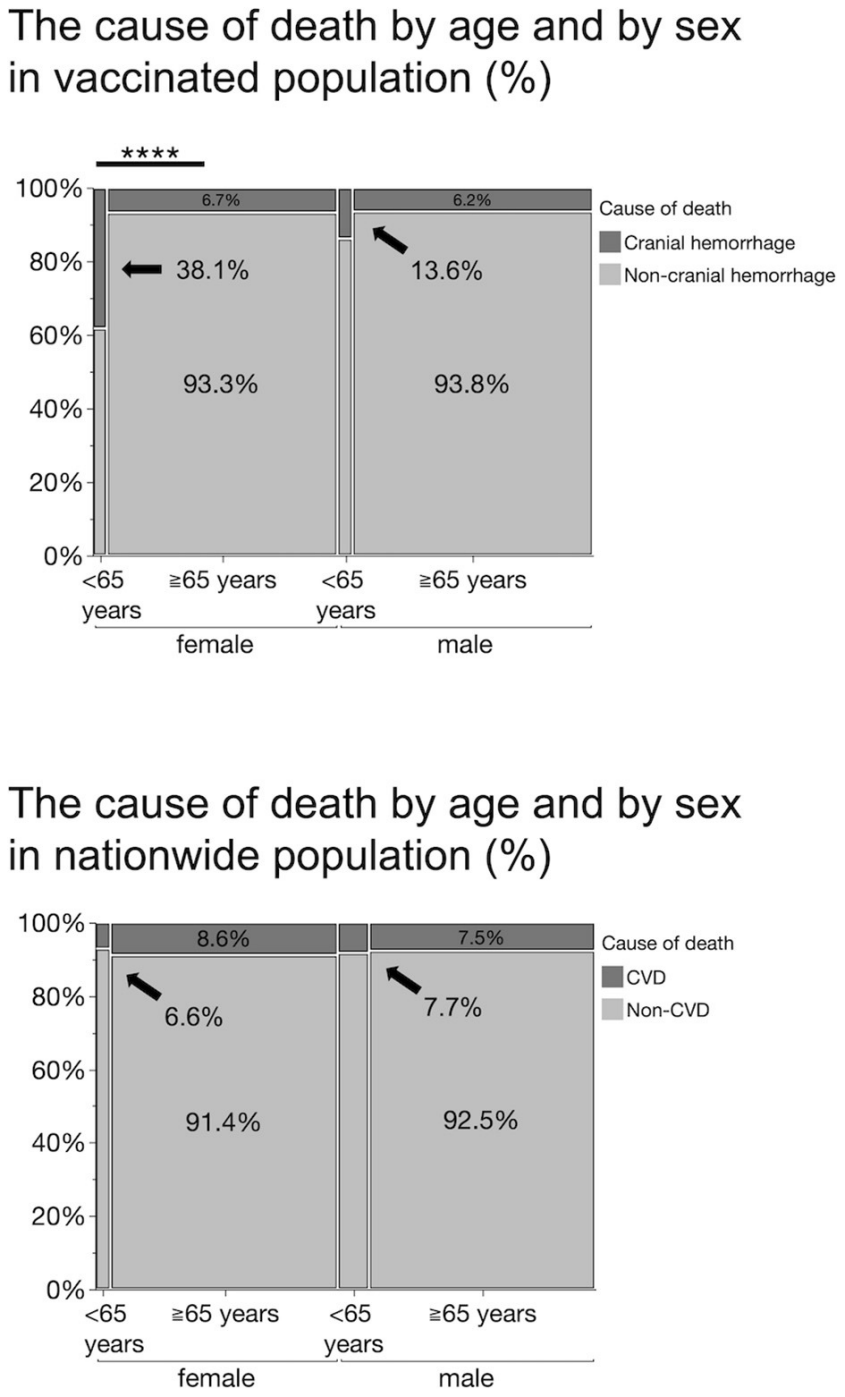
Percentage of the cause of death by age and sex in vaccinated population and in a nationwide population in 2018. Cranial hemorrhages include subdural, subarachnoid, thalamic, cerebellar, brainstem, and cerebral hemorrhages. Cerebrovascular diseases (CVDs) include subarachnoid and intracerebral hemorrhage, cerebral infarction, and other cerebrovascular diseases. The frequency of death by cranial hemorrhage in vaccinated young women (38.1%) is significantly higher than that in the elderly women (6.7%), ****P <0.0001 (*Pearson's chi squared test).

We analyzed the frequency of thrombocytopenia and hemorrhage as a cause of death following the administration of the COVID-19 vaccines. Although thrombocytopenia accounted for only ~1% of the total deaths, bleeding occurred 8.8% of the time. To explore the relationship between sex or age and bleeding, we categorized the cause of death as bleeding, including cranial hemorrhage, cranial hemorrhage alone, and all other causes of death. Cranial hemorrhage accounted for 84.8% of fatal bleeding cases; surprisingly, all bleeding cases in the young were cranial hemorrhage (Supplementary Figure 1). Pearson's chi squared test performed using the data revealed that no sex differences were found in the frequency of bleeding or cranial hemorrhage among deaths (*P* = 0.6814 and *P* = 0.5405, respectively) or among bleeding (*P* = 0.3701) (Supplementary Figure 1). When accounting for age, we found that death from cranial hemorrhage among the young was statistically higher than that among the elderly [frequency of death by cranial hemorrhage: 25.6 vs. 6.5%, $\chi_{\left(1,\;\text{N}=738\right)}^{2}$ = 21.082, *P* <0.0001] (Supplementary Figure 1). Notably, we confirmed that death from cranial hemorrhage among young women was statistically higher than that among elderly women [frequency of death by cranial hemorrhage: 38.1 vs. 6.7%, $\chi_{\left(1,\text{N}=362\right)}^{2}$ = 20.627, *P* <0.0001], while this was not the case in the male population [13.6 vs. 6.2%, $\chi_{(1,\text{N}=376}^{2}$) = 1.838, *P* = 0.1752] (Figure 2). On comparison, the frequency of death due to cerebrovascular diseases (CVD)—including subarachnoid and intracerebral hemorrhages, cerebral infarction, and other CVDs—within the Japanese general population in 2018 (14) did not differ by age or sex, thus emphasizing the potential risk of the vaccine for young women (Figure 2).

In addition, we summarized the case reports of suspected adverse reactions reported by manufacturers and distributors. Of the 75 cases with thrombocytopenia, seven presented with RA and three with ITP (Supplementary Table 1; Supplementary Figure 2).

## Discussion

In this report, we summarized three cases of thrombocytopenia potentially triggered by the administration of BNT162b2 vaccine, at our institution, and review cases of thrombocytopenia and hemorrhage reported by the Japanese MHLW. In an earlier report, Shimazawa and Ikeda focused on 10 fatal cases (five males and five females) after the BNT162b2 vaccination, published by the MHLW on April 23 (15). They warned of a disproportionately high incidence of death from cerebral hemorrhage among Japanese women who received the BNT162b2 vaccine. Consistent with the above observation, we found that the proportion of deaths due to bleeding or intracranial hemorrhage was significantly higher in women, especially in those under 65 years of age. This sex imbalance is incompatible with the mortality data on CVD including subarachnoid and intracerebral hemorrhages, in the National Statistics (Figure 2), as predicted by the authors. In addition, the prevalence of ITP is high among young women in Japan. According to these predictive statistics, CVD accounts for 7.7% of all deaths in men under 65 years of age, compared to 6.6% in women, a significantly lower percentage, whereas our analysis of the BNT162b2 vaccinated population revealed a disproportionately higher incidence of death by bleeding or intracranial hemorrhage in women under 65 years of age than in men.

Cases 1 and 2 in this report also consisted of persistent or temporal thrombocytopenia accompanied by intracranial hemorrhage, suggesting the possibility of thrombocytopenia as a key factor leading to cerebral hemorrhage in these patients. Moreover, this thrombocytopenia may have caused transient hematuria or menorrhagia (16, 17). In Japan, thrombocytopenia has been recognized by regulatory authorities as an adverse reaction to seasonal influenza vaccines. In the Japanese population, immunogenic thrombocytopenia has occurred as an adverse reaction to vaccines against influenza viruses, which, like coronaviruses, are single-stranded RNA viruses. This could be a possible explanation for the association between COVID-19 vaccination and thrombocytopenia-induced autoimmune response against platelets and megakaryocytes, resulting in reticuloendothelial phagocytosis and direct CD8+ T cell lysis (18). Case 3 and four other cases in Supplementary Table 1 suggest the efficacy of therapeutic intervention with corticosteroids, immunosuppressive drugs, or intravenous immunoglobulin (IVIG) for thrombocytopenia. Nevertheless, the spontaneous resolution of the disease in Case 2 and the corticosteroid treatment and IVIG treatment-resistant cases from Supplementary Table 1 indicate that there is no certainty about these treatments. Notably, the three cases in this report share the common features of old age, RA, chronic renal failure or hypertension, and pre-existing mild thrombocytopenia at baseline (11–15 × 10^4^/μL).

The annual incidence of ITP in Japan is 2.2 cases per 100,000 adults per year (19). Among the 35,650,101 persons who received at least one dose of the vaccine, one would expect an average of 783 cases of ITP over the course of a year. Assuming February 17, 2021, as the date of initiation of mass vaccinations for a risk period of 149 days (until July 16, 2021), ~320 cases of ITP are to be expected. In comparison, the MHLW included a minimum of 75 cases of thrombocytopenia. As all of these reports of thrombocytopenia may not be ITP, reports of ITP were not higher than expected among the vaccinated population, consistent with a previous report (5). However, caution should be taken regarding adverse reactions in specific populations, such as young women and RA patients. In this population, vaccine administration is not as advanced as in the elderly population; thus, no conclusions can be drawn yet. As shown in Supplementary Table 1, among the 75 patients with thrombocytopenia, seven patients with RA and three patients with ITP were detected, which represent a higher frequency than that prevalent in Japan (20). Therefore, there is a need to clarify the risk factors for the investigation of thrombocytopenia events following BNT162b2 vaccination. The European League Against Rheumatism states that COVID-19 vaccination is a wise choice for all patients with rheumatic and musculoskeletal diseases (RMDs) and that there is no reason to withhold the vaccines from patients with RMD (21). Patients with RMD are at higher risk of hospitalization due to COVID-19 and may have worse outcomes compared to the general population. Therefore, the American College of Rheumatology and the Asia Pacific League of Associations for Rheumatology strongly recommend vaccination (22, 23). In response to these recommendations, the Japanese College of Rheumatology has also placed RMD patients who are using steroids equivalent to more than 5 mg/day of PSL or immunosuppressive agents, biological agents, or Janus kinase inhibitors at the priority ranking for vaccination (24). A recent report showed that vaccination with BNTb262 resulted in an adequate immunogenic response with an acceptable safety profile in the majority of patients with RMDs (25). However, there is limited but growing evidence of myocarditis in young men following BNT162b2 vaccination (26). Given that the mRNA vaccine is the first of its kind to be commercialized in humans, it is necessary to ensure its short and long-term safety, especially for young patients and those with underlying diseases such as RMD, in order to maximize its efficacy.

This study had some limitations. Studies have shown that the reporting trends of adverse events vary according to the drug's life cycle. Specifically, when a drug is newly introduced, adverse events have a greater chance of being reported due to heightened vigilance of healthcare professionals and may lead to a reporting bias (Weber effect) (27). Thus, healthcare professionals and patients might have been inclined to report adverse events and may systematically link one's death to vaccine administration, especially when death occurs not long after vaccination. Caution must be practiced regarding terminology, as a temporal relationship between vaccine administration and the occurrence of adverse events does not necessarily imply causality. However, the likely overestimation of vaccine-related adverse events and death does not explain the disproportionately high rate of fatal cranial hemorrhage cases in young women. Moreover, an increased amount of time and number of sample sizes are required for the population we focused on in this report in order to observe any tendencies and establish specific guidelines or recommendations regarding vaccine intake by the population. In all three cases, multiple drugs other than the vaccine were administered. To our knowledge, no study has investigated how the vaccine may interact with these drugs, and the possibility of thrombocytopenia caused solely by these medications cannot be excluded. In particular, in clinical trials and post-marketing reports, pancytopenia, leukopenia, neutropenia, aplastic anemia, and thrombocytopenia were identified in patients who received monoclonal antibodies including TNF blockers (28). However, the patient in Case 1 and 2 did not develop fatal thrombocytopenia despite the long-term use of TNF inhibitors. It is thus necessary to compare the frequency of thrombocytopenia in the vaccinated and non-vaccinated groups and perform a longitudinal analysis taking into account the duration of the drug administration. As a more immediate measure, it may be useful to assess post-vaccination platelets counts for populations at risk (i.e., individuals with old age, rheumatoid arthritis, chronic renal failure, hypertension, and pre-existing thrombotic events), given that the majority of the reported cases of thrombocytopenia occurred no longer than 2 weeks after vaccine administration. In fact, without clinical signs, thrombocytopenia is often undetectable unless a blood test is performed. Early medical attention is warranted if bleeding is observed.

As mentioned in the beginning of this report, the Japanese MHLW stated that the possibility that BNT162b2 vaccine was the cause of thrombocytopenia could not be ruled out. The potential association between the BNT162b2 vaccine and thrombocytopenia and bleeding, including intracranial hemorrhage, cannot be discounted and requires further investigation. The clinical data have demonstrated that bleeding is more prevalent in the East Asian population (29). It is therefore clear that the risk-benefit ratio of vaccines would differ from that in the Western population. It is essential to reconsider the risks and benefits based on ethnicity/race in vaccination. For this purpose, the expansion of an established pharmacovigilance system not only in Japan, but also throughout the Asian continent, is urgently required.

## Data Availability Statement

The raw data supporting the conclusions of this article will be made available by the authors, without undue reservation.

## Ethics Statement

Ethical review and approval was not required for the study on human participants in accordance with the local legislation and institutional requirements. Written informed consent for participation was not required for this study in accordance with the national legislation and the institutional requirements.

## Author Contributions

YO and RS conceived and designed the manuscript. YO, RS, and MS-F contributed to data acquisition, interpretation of data, and wrote the manuscript. MN contributed to data acquisition. YO, RS, SY, AS, TKu, TKo, and KA were responsible for clinical care of the patients, as well as for data analysis. YO and RS described the figures with all authors contributing to writing the manuscript and providing advice. All authors contributed to the article and approved the submitted version.

## Conflict of Interest

KA has received research grants from Asahikasei Pharma Corp. and Chugai Pharmaceutical Co. Ltd., and has received speaking fees from AbbVie GK, Astellas Pharma Inc., Chugai Pharmaceutical Co. Ltd., Eisai Co. Ltd., Eli Lilly Japan K.K., GlaxoSmithKline, Janssen Pharma, and Pfizer Japan Inc. The remaining authors declare that the research was conducted in the absence of any commercial or financial relationships that could be construed as a potential conflict of interest.

## Publisher's Note

All claims expressed in this article are solely those of the authors and do not necessarily represent those of their affiliated organizations, or those of the publisher, the editors and the reviewers. Any product that may be evaluated in this article, or claim that may be made by its manufacturer, is not guaranteed or endorsed by the publisher.

## Supplementary Material

Supplementary Figure 1 The vaccinated population and cause of death or bleeding by sex or by age. Cranial hemorrhages include subdural, subarachnoid, thalamic, cerebellar, brainstem, and cerebral hemorrhages. Non-cranial hemorrhages include gastrointestinal, intestinal, intraperitoneal hemorrhages, and conjunctival and alveolar bleeding. ^****^*P* <0.0001 (^*^Pearson's chi squared test).

Supplementary Figure 2 Analysis of Supplementary Table 1. Patients with thrombocytopenia after BNT162b2 vaccine administration. Population analysis of age, sex, distribution of platelet counts, and onset of thrombocytopenia (days after vaccination).

Supplementary Table 1 Patients with thrombocytopenia after BNT162b2 vaccine administration.
