# Supplementary figures and images for: Potential Triggers for Thrombocytopenia and/or Hemorrhage by the BNT162b2 Vaccine, Pfizer-BioNTech
Source: Front Med (Lausanne). 2021 Sep 30;8:751598. doi: 10.3389/fmed.2021.751598 (PMC8514746; doi:10.3389/fmed.2021.751598)

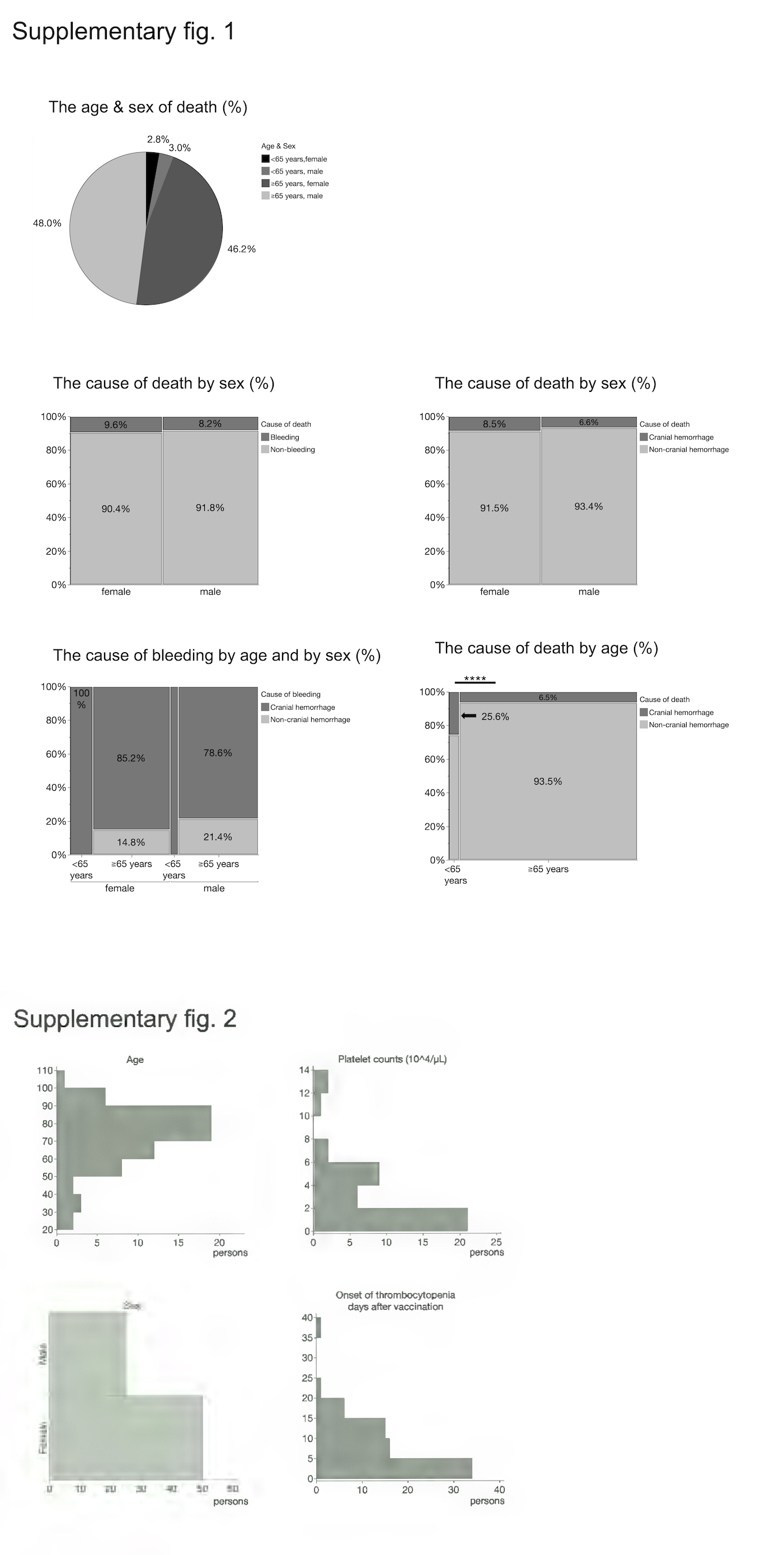

Supplement: Supplementary Figure 1 — The vaccinated population and cause of death or bleeding by sex or by age. Cranial hemorrhages include subdural, subarachnoid, thalamic, cerebellar, brainstem, and cerebral hemorrhages. Non-cranial hemorrhages include gastrointestinal, intestinal, intraperitoneal hemorrhages, and conjunctival and alveolar bleeding. ****P <0.0001 (*Pearson's chi squared test). [file Image_1.TIFF]
